# Diffuse myocardial fibrosis, but not focal fibrosis identified with delayed enhancement, is an independent predictor of LV reversed remodeling in patients with idiopathic non-ischemic cardiomyopathy

**DOI:** 10.1186/1532-429X-15-S1-P109

**Published:** 2013-01-30

**Authors:** Teerapat Yingchoncharoen, Scott D Flamm, Deborah Kwon

**Affiliations:** 1 Cleveland Clinic Foundation, Cleveland, OH, USA

## Background

Diffuse myocardial fibrosis may be a fundamental features of adverse myocardial remodeling in idiopathic non-ischemic cardiomyopathy. As T1-weighted cardiac magnetic resonance (CMR) imaging provides an alternative method of diffuse fibrosis quantification, we sought to assess the association of myocardial T1 value to left ventricular reverse remodeling (LVRR).

## Methods

We performed CMR in 24 patients with idiopathic non-ischemic cardiomyopathy (16 men, mean age 58±11 years) and also in 12 healthy volunteers as control subjects. T1 mapping was performed with post-contrast Look-Locker gradient echo. Baseline echocardiography as well as hemodynamic and metabolic data were collected at the time of CMR. Patients were followed over a median time of 8 months for LVRR which was defined as a left ventricular ejection fraction (LVEF) increase of ≥10 U and a decrease in indexed left ventricular end-diastolic diameter (LVEDD) of ≥10% or indexed LVEDD of < 33 mm/m^2^ at 24 months. A multivariable logistic regression analysis was performed to identify associations with LVRR.

## Results

**Figure 1:**
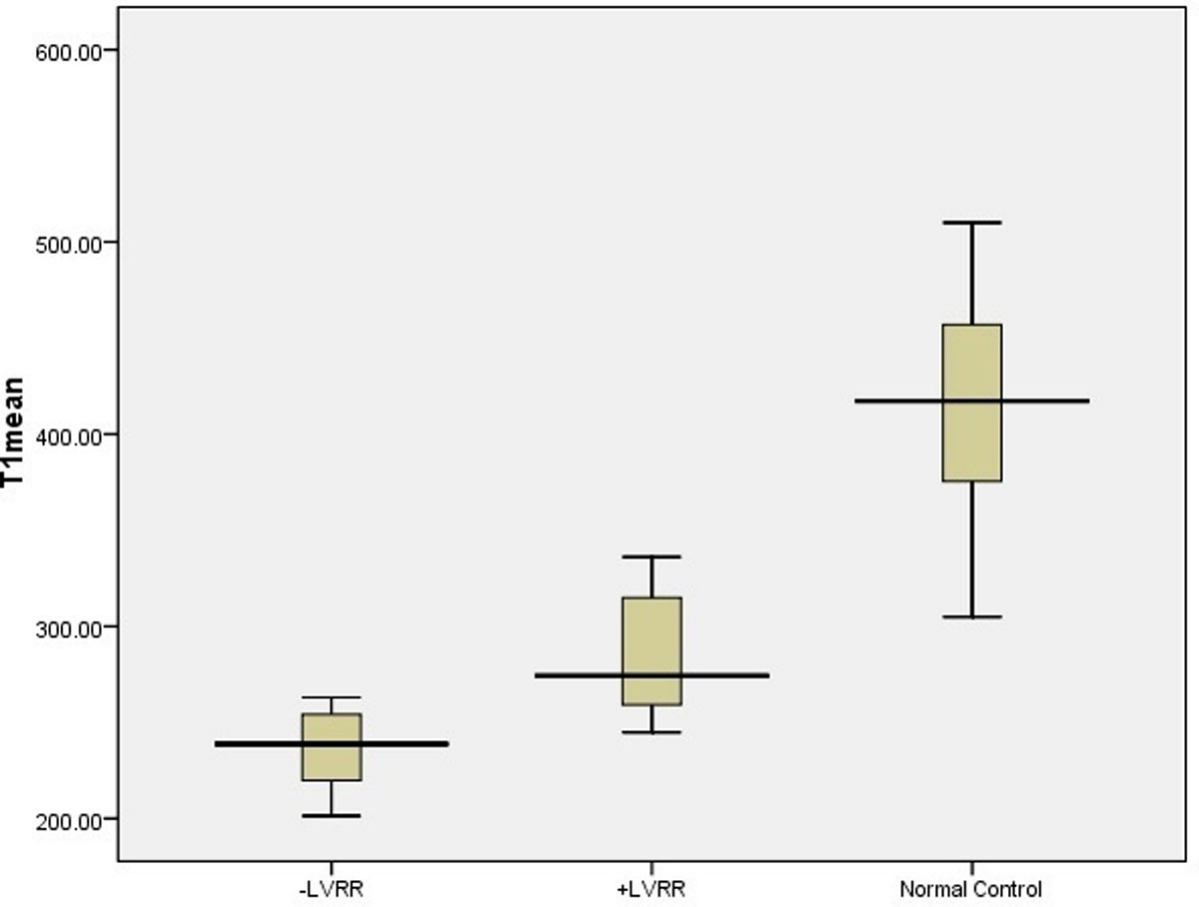
Comparison of myocardial T1 time between patients without LVRR, patients with LVRR and controls.

LVRR was found in 8 patients (33%). Mean T1 value was substantially lower in patients without LVRR (240+26) compared to patients with LVRR (285+35, p=0.002) and healthy controls (413+57, p<0.001) (Figure1). There was no significant difference in T1 value of the non delayed-enhanced myocardium in patients with myocardial scar on delayed-enhancement imaging (264+26) and without scar (263+42, p=0.233). LVRR was associated with baseline T1 value (HR 1.1 [95% CI 1.01-1.19]), independent of LVEF and the presence of myocardial scar on delayed-enhancement imaging (Table 1).

**Table 1:** Multivariate analysis of baseline correlates of LVRR

Variables	HR (95%CI)	P value
Baseline LVEF	0.804 (0.643-1.004)	0.054
Presence of myocardial scar	1.552 (0.091- 26.57)	0.762
Myocardial T1 time	1.098 (1.012-1.192)	0.025

## Conclusions

Post contrast T1 value is a predictor of LV reversed remodeling in patients with idiopathic non-ischemic cardiomyopathy, independent of baseline LVEF and the presence of myocardial scar.

## Funding

None.

